# Acceptance of Home-Based Chlamydia Genital and Anorectal Testing Using Short Message Service (SMS) in Previously Tested Young People and Their Social and Sexual Networks

**DOI:** 10.1371/journal.pone.0133575

**Published:** 2015-07-31

**Authors:** Nicole H. T. M. Dukers-Muijrers, Kevin A. T. M. Theunissen, Petra T. Wolffs, Gerjo Kok, Christian J. P. A. Hoebe

**Affiliations:** 1 Department of Sexual Health, Infectious Diseases and Environmental Health, South Limburg Public Health Service, Geleen, the Netherlands; 2 Department of Medical Microbiology, School of Public Health and Primary Care (CAPHRI), Maastricht University Medical Center + (MUMC+), Maastricht, the Netherlands; 3 Department of Work & Social Psychology, Maastricht University, Maastricht, The Netherlands; Auburn University, UNITED STATES

## Abstract

**Background:**

Control strategies for *Chlamydia trachomatis* (CT) are most effective when targeting people at highest risk. We assessed test acceptance of home-collection test kits offered by short messaging services (SMS) texts, in high-risk young people, i.e. those who had previously tested CT positive (positive indices), or negative reporting more than 3 sex partners (negative indices), and their sexual and social networks.

**Methods:**

Young (16 to 25 years old) heterosexuals who previously tested positive (n=536) or negative (n=536) in our STI clinic received, 3 to 20 months after their initial screening, an SMS inviting them to re-test. They were offered a free home-collection test kit including a genital (men and women) and anorectal (women only) test, and a test kit to pass on to a friend or sex partner (peer). SMS reminders were sent in case of non-response. We assessed proportions of tests requested and returned, peers tested, and positivity. Associations with the individual’s initial screening result and other factors were explored using logistic regression.

**Results:**

Of 1072 people invited to retest, 34.4% (n=369) requested a test. Of these, 55.8% (n=206) retested. Overall, retest participation was higher in positive (22%) than in negative indices (16%) (p<0.001); it was also higher in women and in those aged >22 years. Positivity was 13% and 7% in positive and negative indices, respectively. One in 3 retesters also had a peer tested. Of tested peers (n=87), 84% were friends, 31% were first-time testers, and 7% tested positive.

**Conclusion:**

Acceptance of a relatively low-cost strategy for genital and anorectal testing, i.e. using SMS and home-collection test kits, was highest in individuals who previously tested CT positive suggesting that implementation for this group may be considered. By further including a peer-led testing component, undetected CT positives can be identified in the social networks surrounding a high-risk individual.

## Introduction

Internationally, the number of diagnoses of *Chlamydia trachomatis* (CT) among young people remain high, despite many efforts to reduce and manage the transmission of this infection [1–3]. Testing and treating positives is a key strategy for the interruption of CT spread. Yet testing is precluded by a range of barriers that can be CT-related (e.g. it is an infection largely without symptoms), patient-related (e.g. patients may not feel at risk; may fear the testing method, a positive result, or stigmatization; may have privacy concerns) [4–10], or care-related (e.g. costs, time constraints). Evidence-based strategies designed to overcome some of these barriers and increase testing include the use of postal test kits for home-collection [11–15]. Such postal test kits include self-collected urine, vaginal and anorectal specimens, which are highly acceptable and being used by both men and women [16–18]. The samples derived from these kits can be tested with nucleic acid amplification tests (NAAT), yielding a similar accuracy level to that of clinically derived specimens [19]. Other promising techniques include the use of active recall systems to prompt testing (by letter, phone, email or short message service (SMS)), especially when used in combination with home-collection test kits [12,20,21]. The use of text messages, such as by SMS, has the advantage of being relatively cheap, convenient, and timely. SMS allows providers to interact with patients anywhere and anytime. This is particularly important as patient engagement is key to the effective management of sexual transmitted infections (STIs) [22]. The acceptability of text messages has already been demonstrated in the sexual health context [23]. It has been demonstrated that SMS reminders in combination with home-collection test kits can increase both the number of test requests and returns in young people who have already been invited by letter for testing [14]. Moreover, text messages sent to heterosexual individuals who had previously tested CT positive have been found to increase repeat CT testing at the clinic in some [20, 24], but not in all [25], studies.

SMS technologies and home-collection kits are encouraging developments that can be used to overcome barriers and increase testing. Testing strategies are most effective when directed at CT infected people who are as yet untested and untreated, i.e. the hidden risk groups. Most international guidelines include retesting CT positives, and some also include annual rescreening of high-risk young people, regardless of their initial screening test result [1,2]. Individuals with a previous CT diagnosis show retest positive rates as high as 10%–30% [26, 27]. Unfortunately, a large proportion of these people are not retested, and they remain hidden to care [27, 28]. While home-based testing [15] or active recall by SMS [20,24] have both been shown to increase retesting in some studies, to the best of our knowledge, data are lacking on the combination of SMS and home-based retesting. Contacting people for retesting may provide an excellent opportunity to also reach other hidden CT positives, i.e. in the individual’s social and sexual networks. People in these networks typically show similar high risk, for example with respect to unprotected sex, or sex with a CT positive person [29]. The sexual behaviour, and potentially also the testing behaviour, within social networks has been associated with communication and modelling among friends in these networks [30–33]. Several innovative peer-led intervention programs aiming to reach high risk social and sexual networks have been developed using home-collection and/or e-health technologies [34–37]. Moreover, a few studies have assessed peer-led CT screening using home-based testing [33, 38, 39]. *Loaring et al*. [33], showed that in a small study of 12 women and men, women were potentially more willing to provide their social network with test kits, yielding 34% of kits returned by peers for testing. This peer test return rate was 26% for the sexual partners of 637 diagnosed CT positive persons (general practitioner care) in a study by *Ostergaard et al* [38], while it was only 4.5% for social peers from 67 indices tested in a primary care setting in a study by *Rose et al* [39].

The use of SMS technologies and home-collection test kits in combination with retest strategies and peer-led testing may improve present control strategies and reach more hidden CT positives. We explored exactly this possibility in the current study, in people considered at risk for CT, i.e. young heterosexual men and women who had previously tested CT positive. This group was compared to a group of CT negative young heterosexual individuals at risk for CT, i.e. those who reported having three or more sex partners in the last six months [4]. All of these CT positive and CT negative young people (here called indices) were provided with an extra test kit to pass on to a member of their social or sexual network. Importantly, most strategies designed to increase retesting or peer testing have focused solely on genital infections, missing out on anorectal infections, which occur as frequently in women as do genital CT infections [40–42]. Therefore, in the current study, with the aim of providing a comprehensive screening package, we offered women both genital and anorectal testing.

Findings contribute to existing evidence for the acceptance and yield of comprehensive home-collection test kits combined with SMS reminders on the retesting of CT positives as compared to CT negatives. Moreover, we further investigated test acceptance and yield in their social and sexual networks.

## Patients and Methods

### Study design

We used a controlled observational study design to assess the impact of SMS reminder systems and home-based CT genital and anorectal testing in young heterosexual individuals who had previously (1) screened CT negative or (2) CT positive, called indices, and (3) their social and sexual networks, called peers. The study was approved by the Psychology Research Ethics Board of Maastricht University, ECP-05-09-2012, who waived the need for the active consent of participants. The study was further approved by the Medical Ethical Committee of the University of Maastricht (METC 11-4-108), by using retrospective data originating from standard care (in which one can opt-out for the use of one’s data for scientific research), and by analysing the data anonymously.

### Study population and procedures

The study population was selected from clients attending the STI clinics of the Limburg Public Health Service, which provides approximately 6500 STI consultations annually, offering free examination and treatment. At these outpatient clinics, men who only had sex with women were screened for CT on genital sites only, and women were also screened anorectally on indication of anal sex and/ or symptoms and/or belonging to a high-risk group. The specimens tested consisted of self-collected vaginal swabs or urine (men), and anorectal swabs, and were processed at using nucleic acid amplification assays (NAATs) (PCR, Cobas 4800, Roche, California, USA). For each attendee, contact details (including mobile phone number), age, gender, and sexual orientation were registered.

**Fig 1:**
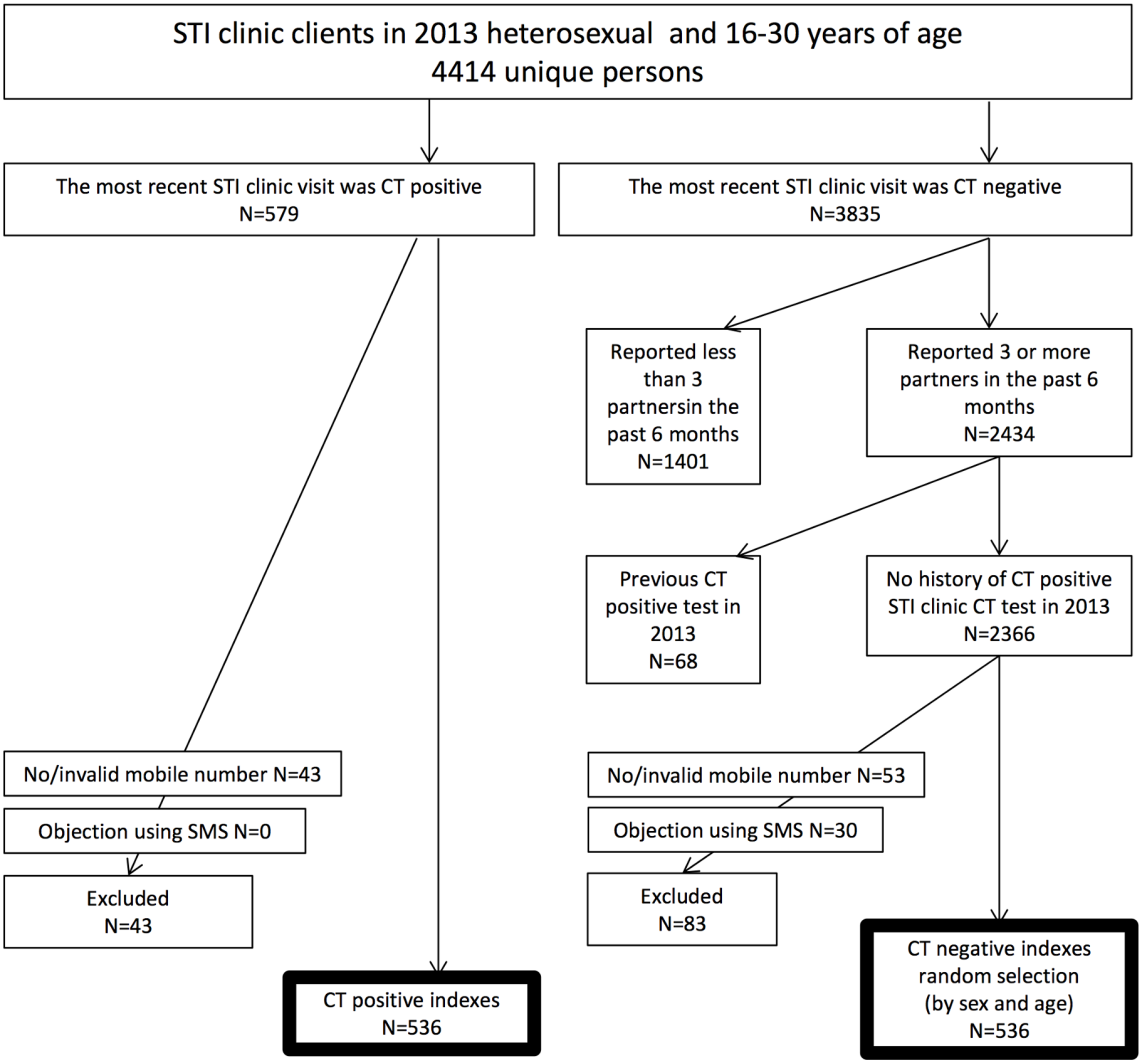
Flow chart of the selection of the study population.

Based on this registry, we selected the most recent test in 2013 from all 4414 unique clinic attendees (indices) who had been screened for CT, were heterosexual, and between 16 and 30 years of age (Fig 1). For the screened CT positive indices, we included those who had a valid mobile phone number registered. As a comparison group, we selected clients who had tested negative on their last CT test, who had reported having three or more partners in the past six months, who had not had a previous positive test in 2013, who did not object to using SMS for communication with the STI clinic, and who had a valid mobile phone number. From this remaining group, a random selection of CT negative indices was made to match the CT positive group in terms of number of males and females, and number of people aged 16–22 and 23–30 years (based on the median age of the CT positives).

The 536 CT positive indices and the 536 CT negative age- and sex- matched indices were sent an SMS containing an invitation to retest by offering a free home-collection CT test kit upon request. The text messages were sent in the period April-July 2014, which was three to 20 months after initial screening. Individuals invited to retest could respond directly, by SMS, or by email (using a smart phone), to provide their contact details so that the testkit could be send to them. If no test was requested following the initial invitation, two extra SMS reminders were sent (after two and four weeks) with a test invitation. When a person requested a test, the test kit was sent within two working days in an unmarked envelope with clear instructions for use. For men, the test kit consisted of a urine sampling kit. For women, the test kit contained a vaginal swab and an anorectal swab. It also contained a pre-paid return envelope addressed to the local laboratory responsible for routine NAAT testing. All men and women who requested a test kit for themselves (i.e. the index) were also sent a test-kit for a friend or sex partner (peer). The test kit for a peer was a separately marked unisex test kit comprising of a urine sampling kit for men, and a vaginal and an anorectal swab for women, also with a prepaid return envelope for testing.

A short questionnaire was also included in the index and peer packages, to be filled in and returned together with the samples. The questionnaire included questions about e.g. sex, date of birth, nationality, intention to give the extra test to a peer (for indices), or who gave them the extra test (for peers). In cases where the test of the index participant was not returned, up to four SMS reminders to return the kit were sent (after two, four, six and eight weeks). Study follow-up to return test kits ended six months after the first SMS invitation was sent. Upon return, the specimens were tested and the test results were communicated via telephone in case of a positive test result and via SMS in case of a negative test result. All CT positives then visited the STI clinic or their general practitioner where they received treatment.

### Statistical analyses

The outcomes investigated: were the proportion of test requests among invited indices, samples returned for testing among both invited indices and among indices who requested a test, positivity among tested indices, and tested peers among indices who requested a test. The outcomes in tested peers further included CT positivity. Proportions were calculated for those who only received an initial invitation SMS and also for those who received extra reminders via SMS. Univariate and multivariate logistic regression were used to identify factors associated with the outcomes (except for the outcome peer positivity, due to low numbers for this outcome). The main focus of interest was the index group, i.e. those who had initially screened CT positive or CT negative. Other factors examined included: nationality (Dutch or non-Dutch), age (≤22 and >22 years), sex of the index, and time elapsed between index initial screening and the SMS invitation to retest (3–12 months and 13–20 months). For the outcomes associated with peer testing, additional factors evaluated included index testing, test result, and intention to pass on a test kit to a peer. A p-value of <0.05 was considered statistically significant. Analyses were performed using the SPSS package version 20 (IBM Inc. Somers, New York, USA).

## Results

The study population comprised 536 CT positive and 536 CT negative young heterosexual individuals who were invited by SMS for retesting via a free home-collection test kit for genital CT and, for women, an additional test kit for anorectal CT (Fig 1). The test kit also contained a unisex test kit for a peer (friend/sex partner). The two groups invited for retest were similar regarding sex (women: 57.5% of negatives and 57.3% of positives), age (both median age 22, interquartile range 20–24 years), and nationality (Dutch nationality: 95.5% of negatives and 97.4% of positives). The median number of days between initial screening and SMS retest invitation was 349 [IQR: 266–440] days for negatives (55.6% between 3–12 months) and 287 [IQR: 201–391] days (69.2% between 3–12 months) for positive indices (p<0.001). At initial screening, all CT negatives and 39.0% (n = 209) of CT positives reported having more than 3 partners in the past 6 months, and 61% (n = 327) of CT positives reported having 3 or less sex partners in the last six months.

### Invitations to retest, reminders, and test requests

**Fig 2:**
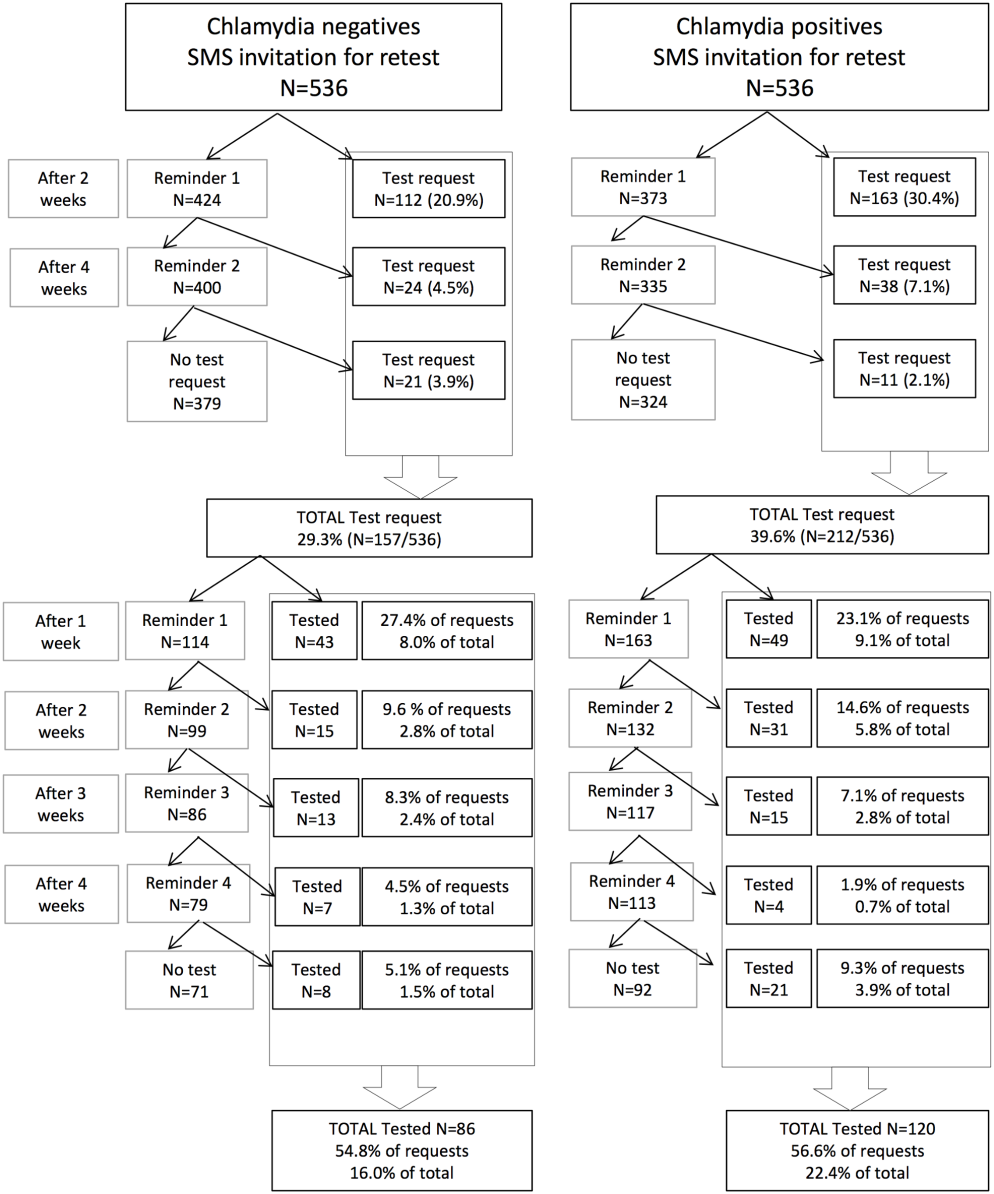
Number of test requests and tests by Chlamydia negative and positive indices.

In total, 369 (34.4%) of all invited young people requested a test kit, i.e. 29.2% of CT negative indices and a significantly higher 39.9% of positive indices (Table 1 and Fig 2). Other factors found to be independently associated with requesting a test included age (those in the older age bracket requested more tests) and sex (females requested more tests). Of the people who requested a test, the majority (71.3% of CT negative individuals and 76.9% of CT positive individuals) did so after the initial invitation SMS; most within a day. The remainder of those who requested a test responded only after one of the reminder SMS has been sent.

**Table 1:** Proportion of test requests, returns, overall testing and *Chlamydia trachomatis* (CT) positivity and associated factors in1072 young people invited by SMS for a retest.

	Proportion of tests requested		Proportion of those who requested a test		Testing		Positivity	
	(1072 invitees)		(n = 369) who also returned the test		among 1072 invitees		among 206 tested invitees	
	% (n)	aOR (95% CI)	% (n)	aOR (95% CI)	% (n)	aOR (95% CI)	% (n)	aOR (95% CI)
Overall	34.4 (369)		55.8 (206)		19.2 (206)		10.2 (21)	
Number of days	Test request-invitation SMS:		Test return-request:		Testing-invitation SMS:			
(median, IQR)	0 days (0–13)		19 days (7–36)		27 days (12–51)			
Initial screening								
Negative	29.3 (157)	1	54.8 (86)	1	16.0 (86)	1	7.0 (6)	1
Positive	39.6 (212)	1.5 (1.2–2.0)*	56.6 (120)	1.1 (0.7–1.7)	22.4 (120)	1.5 (1.1–2.0)*	12.5 (15)	2.0 (0.7–5.6)
Nationality^								
Dutch	34.5 (357)		56.0 (200)		19.3 (200)		10.5 (21)	
Non-Dutch	31.6 (12)		50.0 (6)		15.8 (6)		0 (0)	
Years of age								
16–22	32.8 (219)	1	53.4 (117)	1	17.5 (117)	1	10.3 (12)	1
23–30	37.0 (150)	1.3 (1.0–1.7)*	59.3 (89)	1.5 (0.9–2.2)	22.0 (89)	1.6 (1.2–2.2)*	10.0 (9)	1.0 (0.4–2.8)
Sex								
Male	31.1 (142)	1	43.7 (62)	1	13.6 (62)	1	8.1 (5)	1
Female	36.9 (227)	1.3 (1.0–1.7)*	63.4 (144)	2.3 (1.5–3.5)*	23.4 (144)	2.1 (1.5–2.9)*	11.1 (16)	1.5 (0.5–4.5)
Screening test								
3–12 months ago	37.2 (249)	1.3 (0.9–1.7)#	57.8 (144)	1.1 (0.7–1.8)	21.5 (144)	1.4 (0.9–1.9)#	9.7 (6)	0.9 (0.3–2.5)
13–20 months ago	29.8 (120)	1	51.7 (62)	1	15.4 (62)	1	10.4 (15)	1

*p<0.05,

^#^ p<0.10

CI: Confidence Interval; aOR: adjusted Odds Ratio, meaning that risk estimates were adjusted when applicable for initial screening result of the index, age of the index, sex of the index, timing of the screening test of the index

^^^nationality was not included as a factor as nearly all participants had Dutch nationality

### Returning kits for laboratory testing, and reminders

In total, 206 (55.8%) of all young people who requested a kit returned it to the laboratory for testing. This proportion was similar between index groups with 54.8% for CT negatives and 55.6% for CT positives. The only factor found to be associated with returning the test kit was sex, with females returning significantly more test kits than males (Table 1). Of those who returned the test, about half (50.0% of CT negative indices and 40.8% of CT positive indices) returned the test within two weeks. The rest did so after having been sent one or more SMS reminders to return the test kit.

### Overall retesting and associated factors

In total, taking into account all persons invited to retest by SMS, 16.0% of CT negatives and a significantly higher proportion (22.4%) of CT positives were retested for CT (Table 1). Other factors independently associated with retesting were sex (with more females retesting than males), and age (with those being in the older age bracket more likely to retest than those in the younger age bracket).

### Positivity upon retesting in indices

In total, 21 indices (10.2%) tested CT positive upon retesting. Positivity did not differ between CT positive and negative indices, or between other studied factors (such as age and sex) (Table 1). The majority of positives (71.4%, n = 15) had received extra SMS reminders to request/return the test kit in addition to the initial invitation SMS.

In women, positivity for genital CT was 6.9% (10/144); 86.8% of these women (n = 125) also tested anorectally, of which 11.2% (14/125) were anorectal CT positive. Of anorectal cases, 42.9% (6/14) were single infections (i.e. not concurrent with genital CT). For most (n = 12) of these anorectal CT positive women, it was the first time they had been anorectally tested. Five of these women with a single anorectal CT were again tested at the STI clinic treatment visit to rule out the possibility that there had been a home-collection sample mix-up and that the genital region was sampled instead of the anorectal region. All these women again tested positive for single anorectal CT.

### Index-related factors associated with peer testing

The proportion of peers that tested was similar across the two CT index groups who requested a test for themselves (Table 2). Peer testing was associated with the sex of the index (female indices were more likely than male indices to have a peer tested), whether the index was screened more recently, and whether an index retested. Further, in retested indices, peer testing was higher (48.2%; 43/110) for indices who expressed the intention to pass on the extra peer test kit. Peer testing was only 18.8% when no such intention was reported by the retested index; peer testing was 9.8% when the index had not retested (Table 2).

**Table 2:** *Chlamydia trachomatis* (CT) peer testing and associated factors among 369 indices who requested a CT test for themselves and got an extra test for a peer, and positivity among tested peers.

	Peer testing		Positivity among
	% (n)	aOR (95% CI)	87 tested peers % (n)
Overall	23.3 (87)		6.9 (6)
Initial screening result index			
Negative	23.6 (37)	1	5.4 (2)
Positive	23.1 (50)	1.0 (0.5–1.5)	8.0 (4)
Nationality index^			
Dutch	23.8 (85)		7.1 (6)
Non-Dutch	16.7 (2)		0 (0)
Age index (years)			
16–22	22.8 (50)	1	10.0 (5)
22–30	24.7 (37)	1.2 (0.7–2.0)	2.7 (1)
Sex index			
Male	14.8 (21)	1	0 (0)
Female	29.1 (66)	1.8 (1.0–3.3)*	9.1 (6)
Screening test index			
3–12 months ago	27.3 (68)	1.9 (1.0–3.5)*	7.4 (5)
13–20 months ago	15.8 (19)	1	5.3 (1)
Index re-tested			
No	9.8 (16)	1	6.3 (1)
Yes	34.5 (71)	4.3 (2.4–7.9)*	7.0 (5)
*Among 206 retested indices*			
Index retest result			
Negative	34.1 (63)	1	6.3 (4)
Positive	38.1 (8)	1.1 (0.4–3.1)	12.5 (1)
Intention of index to pass on the peer test #			
Don’t know yet	18.8 (16)	1	0 (0)
Yes to social network member	47.6 (40)	3.7 (1.9–7.3)*	10.3 (4)
Yes to sexual network member	57.7 (15)	6.9 (2.5–19.2)*	7.1 (1)

CI: Confidence Interval; aOR: adjusted Odds Ratio, meaning that risk estimates were adjusted when applicable for initial screening result of the index, years of age of the index, sex of the index, timing of the screening test of the index, and whether the index retested.

*p<0.05

^^^nationality was not included as a factor as nearly all participants had Dutch nationality

^#^ reported at retesting of the index

Of the 206 indices that retested themselves, 53.4% (n = 110/206) intended to pass on the extra test kit to a peer; this proportion did not differ between groups defined by indices’ sex, age, or time elapsed since initial index screening (data not shown).

Among the 110 indices who had the intention to pass on the extra test, 76.4% (n = 84) mentioned that they would pass it on ‘to a friend’ and 23.6% (n = 26) indicated that they would pass it on ‘to a sex partner’. Intention to pass on ‘to a friend’ was higher in female indices (87.0%) than male indices (51.5%; p<0.001).

### Characteristics of tested peers and peer-index pairs

Of the total number of 87 peers tested for CT; 62 (71.3%) peers did so after their index had received a reminder SMS to request and/or return the test kit. About a third of tested peers had never been tested for CT before, and over two-thirds of the peer-index pairs were concordant in their age group, sex, and nationality (Table 3). Most peers received their test from a best friend (39.1%, n = 34), friend (42.5%, n = 37), or more distant friend (4.6%, n = 4), while a smaller number got the test from their steady sex partner 12.6% (n = 11) or regular casual partner (1.1, n = 1). No one received the test from a casual partner.

**Table 3:** Characteristics and positivity of 87 tested peers, concordance with the 87 indices that gave them the test.

		Positivity in tested peers
	% (n)	%^ (n)
Peer Age (years)		
16–21 years	32.2 (28)	7.1 (2)
22–34 years	67.8 (59)	6.8 (4)
Peer-index pairs: concordance age		
Both age ≤22 years	27.6 (24)	8.3 (2)
Both age >22 years	37.9 (33)	3.0 (1)
Discordant age	34.56 (30)	10.0 (3)
Peer sex		
Female sex	78.3 (69)	7.2 (5)
Male sex	20.7 (18)	5.6 (1)
Peer-index pairs: concordance sex		
Both female sex (friends)	64.4 (56)	8.9 (5)
Both male sex (friends)	6.9 (6)	0 (0)
Discordant sex (friends)	12.6 (11)	9.1 (1)
Discordant sex (sex partners)	16.1 (14)	0 (0)
Peer nationality		
Dutch nationality	90.8 (79)	7.6 (6)
Non-Dutch nationality	9.2 (8)	0 (0)
Peer-index pairs: concordance nationality		
Both have Dutch nationality	88.5 (77)	7.8 (6)
Both have non-Dutch nationality	1.1 (1)	0 (0)
Discordant nationality	10.3 (9)	0 (0)
Peer history of CT testing		
Never tested	31.0 (27)	11.1 (3)
Previously tested	69.0 (60)	5.0 (3)

^^^percentages were calculated excluding missing information for 18 indices that did not provide information as they were untested

Notably, all but one of the tested peers from indices that intended to pass the test on ‘to a friend or did not know yet to whom to pass on the peer test’ had indeed received the test from a friend. However, 28.6% of tested peers from indices that intended to pass on the test ‘to a sex partner’ reported that they had actually received the test from a friend.

### Positivity of the peers and peer-index pairs

In total, six peers tested positive for CT (6.9%). Of all six positives (100%), the index had received an extra reminder SMS to request and/or return the test kit. Three of these positives had never been tested before. Positivity did not significantly differ by initial screening test result of the index or by other studied factors in Chi-square analyses (Table 3). Overall positivity in female peers was 7.7%. Genital positivity in female peers was 5.8% (4/69) and in the 58 (84.1%) women that were also anorectally tested positivity was 6.9% (n = four) for anorectal CT; one anorectal CT was a single infection (i.e. not concurrent with genital CT). All positives (one man and five women) got their test from a female friend.

## Discussion

To increase *Chlamydia trachomatis* testing in young heterosexual people at risk for CT, we combined several promising care methods including home-collection test kits for anorectal and genital CT testing, active recall by SMS technology, reminder systems, and peer-led testing. Home-collection testing following SMS invitation was moderately accepted, and rates of home-collection testing were higher in previously tested CT positives (22%) than CT negatives (16%). Peer-led testing was similar (23%) across positive and negative indices, and as a result of implementing our system, several never-tested high-risk peers were reached. Acceptance and yield of anorectal CT testing in women–also outside the care setting- was high. Current standard care may well benefit from implementing and integrating these different components to existing CT control strategies.

Individuals with a previous CT diagnosis and those reporting behavioural risk (e.g. a high number of partners, three or more partners in the current study) are well known risk groups for CT, and are specifically targeted in current testing guidelines [1,2]. Acceptance of CT retesting via home-collection test kits was highest in CT positive indices. Prior CT positives are considered more likely to be motivated to get rescreened [43]. Still, test return rates and peer testing rates were similar in CT positive indices as compared to high-risk CT negative indices. Notably, in CT positive indices, the number of partners reported at the time of initial screening was not associated with subsequent test requests, test returns or peer testing. Nevertheless, CT negative indices had a lower acceptance of home-collection testing and about half the number of new CT positive diagnoses as compared to positive indices. Thereby the impact of SMS invitations on reaching hidden CT positives is lowest in CT negative indices, despite them reporting having more than three partners.

Overall, the test rate can be considered moderate when compared with other studies that have used active recall or home-collection testing, with testing rates in these studies ranging from 3–48% [11]. It should be noted that direct comparisons are difficult to make, as retest rates differ by sex, age and the approach taken. A comparison with historical data in the period 2006–2010 from our own clinic (a period when no active recall strategies were applied) showed in CT positive indices higher retesting rates (33% versus 22% in the present study), and positivity rates (19% versus 13% in the present study) [27]. Still, a thorough comparison is difficult to make, as rates may also be influenced by other factors potentially associated with retesting, such as time (e.g. testing policy), and sexual orientation (the current study only includes heterosexual individuals). It is unknown to what extent retesting rates were underestimated in the current study, as a proportion of patients may have undergone screening of their own volition, either at our clinic or at other health services. Previous studies have demonstrated a lower positivity rate in those who retest following active recall as compared to retesting without active recall, and it has been suggested that interventions to encourage rescreening may (also) reach patients at lower risk of re-infection [11].

People at risk for CT also include the sexual and social networks surrounding a person at risk, and these networks were effectively targeted in the current study. It is encouraging to note that young people who requested a test for themselves (following the offer of a home-collection test kit by SMS) were also quite often willing to pass on an extra test to their peers. This peer test was sent to all indices who requested a test for themselves (indices did not actively request an extra peer test). While the index’s test result did not seem to influence peer testing, peer testing was associated with the index’s own testing (35% of peers tested when the index was tested). It was also associated with the intention of the tested index to pass on the test to a peer (peer testing was 46% when the intended peer was a friend, and 54% when the intended peer was a sex partner). Our study therefore demonstrates the potential these methods show in terms of reaching social and sexual peers for testing, confirming the findings of earlier studies [33,38]. Strikingly, most (85%) of the tested peers were friends rather than sex partners, and CT positivity was highest among these peer friends (8%). Even when indices intended to pass on the peer test to a sex partner, a quarter of their tested peers turned out to be friends, showing that indices tended to switch from the intended sexual to social peer type but not vice versa. Bearing in mind that a third of peers had never been tested before, peer-led CT testing appears to be a valuable tool that can be used to reach hidden young people at risk for CT, especially in the social networks. This highly promising method could be incorporated into existing CT testing strategies. Actual peer-testing may have been even higher, as we were unable to determine how many of the kits provided to indices were not passed on to peers. Also we have not assessed what the reasons behind (not) passing on tests or (not) retesting might be. An accompanying publicity campaign about the kits could potentially increase both awareness and use of the kits by peers [39]. The rate of kit use might also be increased if different options for requesting (e.g. via SMS, internet, telephone and pick up from the clinic) and returning (e.g. dropping off at the clinic) the test kits were available, depending on the diverse needs and preferences of different risk groups, as suggested by Smith *et al* [44].

In line with the literature, women were more likely than men to be retested using home-collection test kits [15,21] or have their peers tested [33,38,39]. In another study, women felt able to discuss home-collection CT testing among their close friends, whereas males experienced embarrassment and difficulty discussing and sharing home-collection testing amongst their social network members [33]. In the current study, as many as 22 of the (in total) 27 CT positive diagnoses originated from female indices. Although test acceptance in men is generally lower, it is notable that the intention to pass on the extra test to a peer was similar in tested male and female indices. Also, peer testing rates were similar for retested male indexes (38%) and for retested female indices (27%) (data not shown). Still most of the tested peers were women. It is unknown why male peers are less frequently tested. It may be that indexes are less likely to pass on their test to a male peer, or it may be that male peers do receive a kit but for some reason do not test. More research is needed to understand these reasons for not testing and to improve care strategies targeted at men. Other methods evaluated in the current study include the use of extra SMS reminders, which were shown to positively influence the numbers of tests and diagnoses made, in line with previous findings [14]. In general, SMS is cheap, can be largely automated, but still allows prompt and personal communication. Our experience was that in several cases, invitees phoned back to check for confidentiality and to ensure that the invitation was ‘real and reliable’. We recommend having a nurse available to answer such calls, whereas the SMS process itself can be fully automated. Importantly, this is the first time that acceptance of anorectal CT testing in women has been demonstrated outside the care setting [40–42]. In line with studies investigating women attending STI clinics, anorectal CT positivity in women outside the clinic setting is at least as high as genital CT positivity. While anorectal infections are largely concurrent with genital CT, they also do occur as single infections [40–42].

Although different aspects of our methodology were shown to be successful, there is room for improvement in terms of how they are implemented. For example, the loss of test kits that were sent out but not returned is a waste of resources. In our current routine care, we no longer immediately send out the extra peer test, but only do so after the index has expressed his or her intention to pass on the peer test kit. To decrease loss of test kits and improve return rates, we also added a fifth and sixth SMS reminder. It should be noted that the test return rate of the indices is in line with the overall return rate in a systematic review of studies that also used home-collection test kits for the active recall and retesting of Ct positives [21].

Our study also has several limitations. The first is that we did not have a randomized control group who received standard care only. Consequently we do not know whether our combined approach using SMS and home-based testing is more effective than an approach with no active recall. The second limitation is that, as mentioned above, all participating indices were provided with an extra test kit to pass to a peer without first ascertaining their willingness to do so. Had these extra test kits for peers only been provided to indices who indicated that they were willing to pass on an extra test kit to a peer, return rates might well have been higher.

In conclusion, acceptance of a relatively low cost, feasible strategy for genital and anorectal CT retesting—i.e. using SMS technology, reminder systems and home-collection test kits—is higher in previously CT positive than negative indices. Those who retest are willing to provide tests to their sex partners, and especially friends. By including this peer-led testing component, undetected CT positives can be identified in the social networks surrounding a high-risk individual. However, patients at risk for CT may choose different ways in which to test and it is important for programs to provide different options offering integrated CT control strategies.
